# Polymer films doped with fluorescent sensor for moisture and water droplet based on photo-induced electron transfer†

**DOI:** 10.1039/d1ra02673a

**Published:** 2021-05-10

**Authors:** Takuma Fumoto, Saori Miho, Yuta Mise, Keiichi Imato, Yousuke Ooyama

**Affiliations:** Department of Applied Chemistry, Graduate School of Engineering, Hiroshima University 1-4-1 Kagamiyama Higashi-Hiroshima 739-8527 Japan yooyama@hiroshima-u.ac.jp +81-82-424-5494

## Abstract

Anthracene-(aminomethyl)phenylboronic acid pinacol ester (AminoMePhenylBPin) OF-2 acts as a PET (photo-induced electron transfer)-type fluorescent sensor for determination of a trace amount of water: the addition of water to organic solvents containing OF-2 causes a drastic and linear enhancement of fluorescence emission as a function of water content, which is attributed to the suppression of PET. Indeed, detection limits (DLs) for OF-2 were as low as 0.01–0.008 wt% of water in solvents, that is, the PET method makes it possible to visualize, detect, and determine a trace amount of water. Thus, in this work, in order to develop fluorescent polymeric materials for visualization and detection of water, we have achieved the preparation of various types of polymer films (polystyrene (PS), poly(4-vinylphenol) (PVP), polyvinyl alcohol (PVA), and polyethylene glycol (PEG)) which were doped with OF-2, and investigated the optical sensing properties of the OF-2-doped polymer films for water. As-prepared OF-2-doped polymer films initially exhibited green excimer emission in the PET active state, but blue monomer emission in the PET inactive state upon exposure to moisture or by water droplet. Moreover, it was found that the OF-2-doped polymer films show the reversible fluorescence properties in the dry–wet process. Herein we propose that polymer films doped with PET-type fluorescent sensors for water based on a fluorescence enhancement (turn-on) system are one of the most promising and convenient functional materials for visualizing moisture and water droplets.

## Abstract 2

Polymer films doped with a photo-induced electron transfer (PET)-type fluorescent sensor exhibit green excimer emission in the PET active state, but blue monomer emission in the PET inactive state upon exposure to moisture.

## Graphical abstract

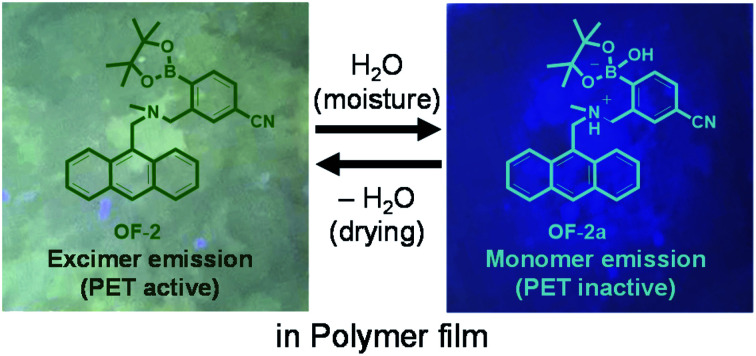

## Introduction

**Fig. 1:**
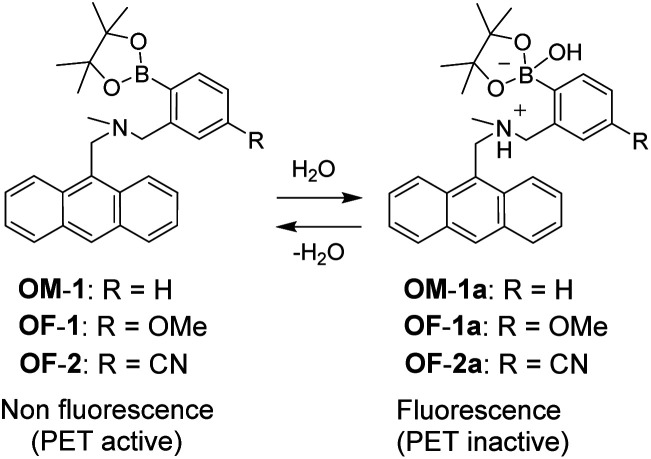
Proposed mechanisms of PET-type fluorescent sensors OM-1, OF-1, and OF-2 for detection of water in organic solvents.

Needless to say, a novel coronavirus, severe acute respiratory syndrome coronavirus 2 (SARS-CoV-2) that causes the Coronavirus Disease 2019 (COVID-19), dramatically changed the world to give people a sense of fear of death. Infectious viruses are generally released into the atmosphere through droplet spread from coughing and sneezing by an infected person. Thus, the infection route from an infected person to an uninfected person is predominately due to the droplet. Actually, face shields made of polyester or polycarbonate films and partitions made of acrylic resin are commercially available for reducing the risk of droplet infection. Therefore, if we can visually confirm the droplet on the face shields and partitions, this allows us to accurately remove the viruses by wiping away the droplet. However, because the virus-containing droplet is generally 5 μm or more, it is practically difficult for us to visually confirm the droplet. Meanwhile, over 90% of the droplet is composed of water, and thus techniques and methods capable of visualizing water are undoubtedly useful for detecting the virus-containing droplet. Optical methods utilizing colorimetric and fluorescent sensors for visualization as well as detection and quantification of water in samples and products, such as solutions, solids, and gases or water on substrate surfaces have been of considerable scientific and practical concern in recent years, because of not only fundamental studies in photochemistry, photophysics, and analytical chemistry, but also their potential applications to environmental and quality control monitoring systems and industry.^1,2^ To date, various kinds of organic fluorescent sensors and polymers for the determination of water content based on ICT (intramolecular charge transfer),^3–5^ ESIPT (excited state intramolecular proton transfer),^6,7^ PET (photo-induced electron transfer),^8–10^ or solvatochromic properties^11^ have been designed and synthesized. The optical sensing properties of these fluorescent sensors for the detection and quantification of water content were investigated from the viewpoints of the relationship between ICT, ESIPT, or PET characteristics and the intermolecular interaction of the sensors with water molecules. As a result, it was found that most of the previous fluorescent sensors for the water content determination, including fluorescent conjugated polymers^12^ and organic fluorescent dyes with ICT and ESIPT characteristics, are based on a fluorescence quenching (turn-off) system, that is, the fluorescence intensity of the sensors decreases as a function of water content in organic solvents. However, this fluorescence quenching system makes it difficult to detect a trace amount of water. In contrast, a fluorescence enhancement (turn-on) system, where the fluorescence intensity increases with an increase in water content in organic solvents, is useful for the visualization, detection, and quantification of a trace amount of water in organic solvents. In particular, the fluorescence enhancement system based on PET-type fluorescent sensors can detect reversible changes in their immediate environment, *i.e.*, water content, through the reversible intermolecular interactions between the sensors and water molecules. Actually, during the past decade, we have designed and developed anthracene-(aminomethyl)phenylboronic acid pinacol esters (*e.g.*, OM-1, OF-1, and OF-2)^9*a*,*d*^ as PET-type fluorescent sensors for the determination of a trace amount of water (Fig. 1).^8–10^ The PET takes place from the nitrogen atom of the amino moiety to the photoexcited fluorophore (anthracene) skeleton in the absence of water, leading to fluorescence quenching. The addition of water to organic solvents containing the PET-type fluorescent sensors causes a drastic and linear enhancement of fluorescence emission as a function of water content, which is attributed to the suppression of PET; that is, the nitrogen atom of the amino moiety is protonated or strongly interacts with water molecules, leading to the formation of the PET inactive species such as OF-2a.^9*d*^ Indeed, detection limits (DLs) for OF-2 were as low as 0.01–0.008 wt% of water in solvents. Thus, the PET method makes it possible to visualize, detect, and determine a trace amount of water. Consequently, fluorescent sensors for water are one of the most promising functional materials contributing to not only post-COVID-19 society, but also Sustainable Development Goals (SDGs), which has been adopted by all United Nations Member States in 2015 and provides a shared blueprint for peace and prosperity for people and the planet now and in the future. In fact, some fluorescent conjugated polymers^12^ and ICT-type^5^ or ESIPT-type^7^ fluorescent sensor-doped polymer films for water that are based on a fluorescence quenching (turn-off) system have been prepared. However, to the best of our knowledge, there are no report on PET-type fluorescent polymers and PET-type fluorescent sensor-doped polymer films for water.

Thus, in this work, in order to develop fluorescent polymeric materials for visualization and detection of water, we have achieved the preparation of various types of polymer films (polystyrene (PS), poly(4-vinylphenol) (PVP), polyvinyl alcohol (PVA), and polyethylene glycol (PEG)) which were doped with PET-type fluorescent sensor OF-2, and investigated the optical sensing properties of the OF-2-doped polymer films for water. As-prepared OF-2-doped polymer films initially exhibited green excimer emission in the PET active state, but blue monomer emission in the PET inactive state upon exposure to moisture or by water droplet. Moreover, it was found that the OF-2-doped polymer films show the reversible fluorescence properties in the dry–wet process. Herein we propose that polymer films doped with fluorescent sensors for water are convenient functional materials for visualizing the virus-containing droplet.

## Results and discussion

**Fig. 2:**
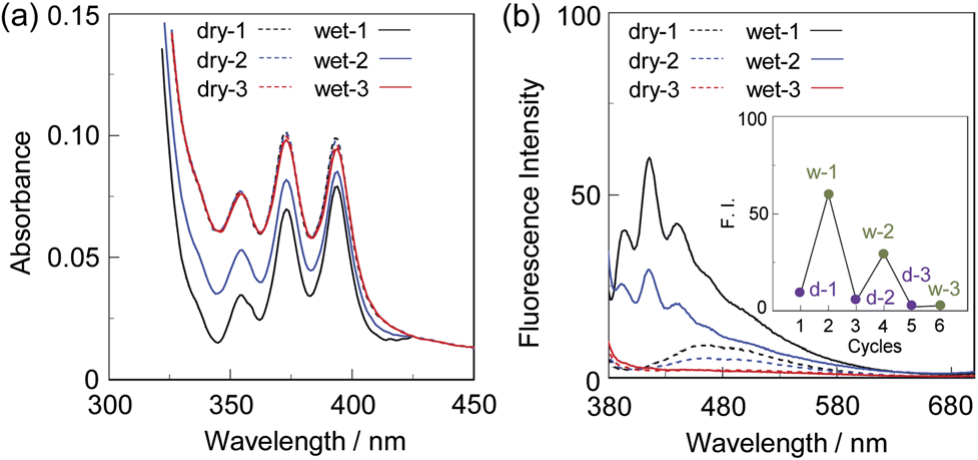
(a) Photoabsorption and (b) fluorescence spectra (*λ*^ex^ = 366 nm) of spin-coated OF-2 film before (in dry process) and after (in wet process) exposure to moisture. For photoabsorption spectra, baseline-correction has been made to be the same absorbance at 425 nm for all the spectra. Inset in (b) shows reversible switching of fluorescence intensity at around 470 nm in dry process and at 415 nm in wet process of spin-coated OF-2 film.

First, in order to investigate the optical properties of OF-2 in the aggregate state, the spin-coated OF-2 film was prepared on a glass substrate, and the photoabsorption and fluorescence spectra of the spin-coated OF-2 film before and after exposure to moisture were repeatedly measured several times (Fig. 2). As with the case of OF-2 in absolute acetonitrile (Fig. S1, ESI†),^9*d*^ the as-prepared spin-coated OF-2 film (in dry process) shows a vibronically-structured photoabsorption band in the range of 300 nm to 400 nm originating from the anthracene skeleton (Fig. 2a). The photoabsorption spectral shape of the spin-coated OF-2 film did not undergo appreciable changes upon exposure to moisture, as with the case of OF-2 in acetonitrile that contained various concentrations of water, although a slight change in absorbance of the spin-coated film during the repeated cycles was observed due to the disturbance of baseline in the photoabsorption spectra. In the corresponding fluorescence spectra, OF-2 in absolute acetonitrile exhibits a feeble but vibronically-structured fluorescence band with a fluorescence maximum wavelength (*λ*^fl^_max_) of 415 nm in the range of 400 nm to 500 nm in the PET active state, which is attributed to the monomer emission originating from the anthracene skeleton. The enhancement of the monomer emission band was observed upon addition of water to the acetonitrile solution of OF-2, which is due to the suppression of PET (the PET inactive state) (Fig. S1, ESI†).^9*d*^ On the other hand, the as-prepared spin-coated OF-2 film shows a feeble and broad fluorescence band in the range of 400 nm to 600 nm, which is assigned to the excimer emission originating from the anthracene skeleton in the PET active state (Fig. 2b). Interestingly, the spin-coated OF-2 film underwent a change in the fluorescence spectra upon exposure to moisture (in wet process), which caused the vibronically-structured monomer emission (*λ*^fl^_max_ = 415 nm) arising from the PET inactive state. Moreover, it was found that when the spin-coated OF-2 film after exposure to moisture was dried in the atmosphere, the photoabsorption and fluorescence spectra showed the original spectral shapes before exposure to moisture. Thus, for the spin-coated OF-2 film, the reversibility of the fluorescence intensity between the excimer and monomer emissions in the dry–wet (moisture) process was investigated (Fig. 2b inset). The dry–wet cycle shows that the monomer emission was not observed in the third wet process. The poor reversibility of the fluorescence intensity of the spin-coated OF-2 film between the excimer and monomer emissions may be attributed to destruction of the film during the dry–wet process.

**Fig. 3:**
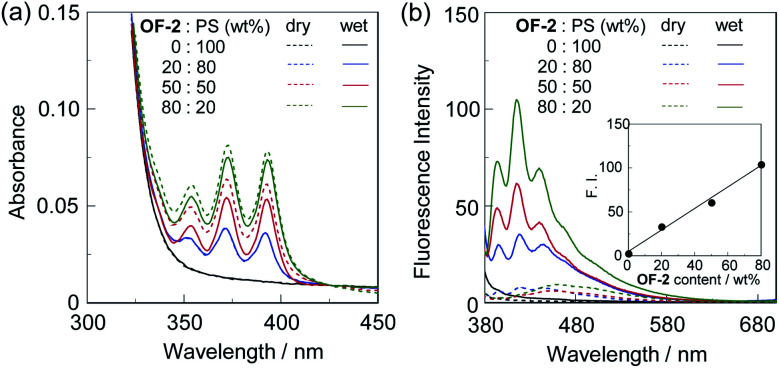
(a) Photoabsorption and (b) fluorescence spectra (*λ*^ex^ = 366 nm) of spin-coated PS films doped with various concentrations (20, 50, and 80 wt%) of OF-2 before (in dry process) and after (in wet process) exposure to moisture. For photoabsorption spectra, baseline-correction has been made to be the same absorbance at 425 nm for all the spectra. Inset in (b) shows fluorescence peak intensity at 415 nm (*λ*^ex^ = 366 nm) in wet process of OF-2-doped PS films as a function of the concentration of OF-2 in the PS films.

**Fig. 4:**
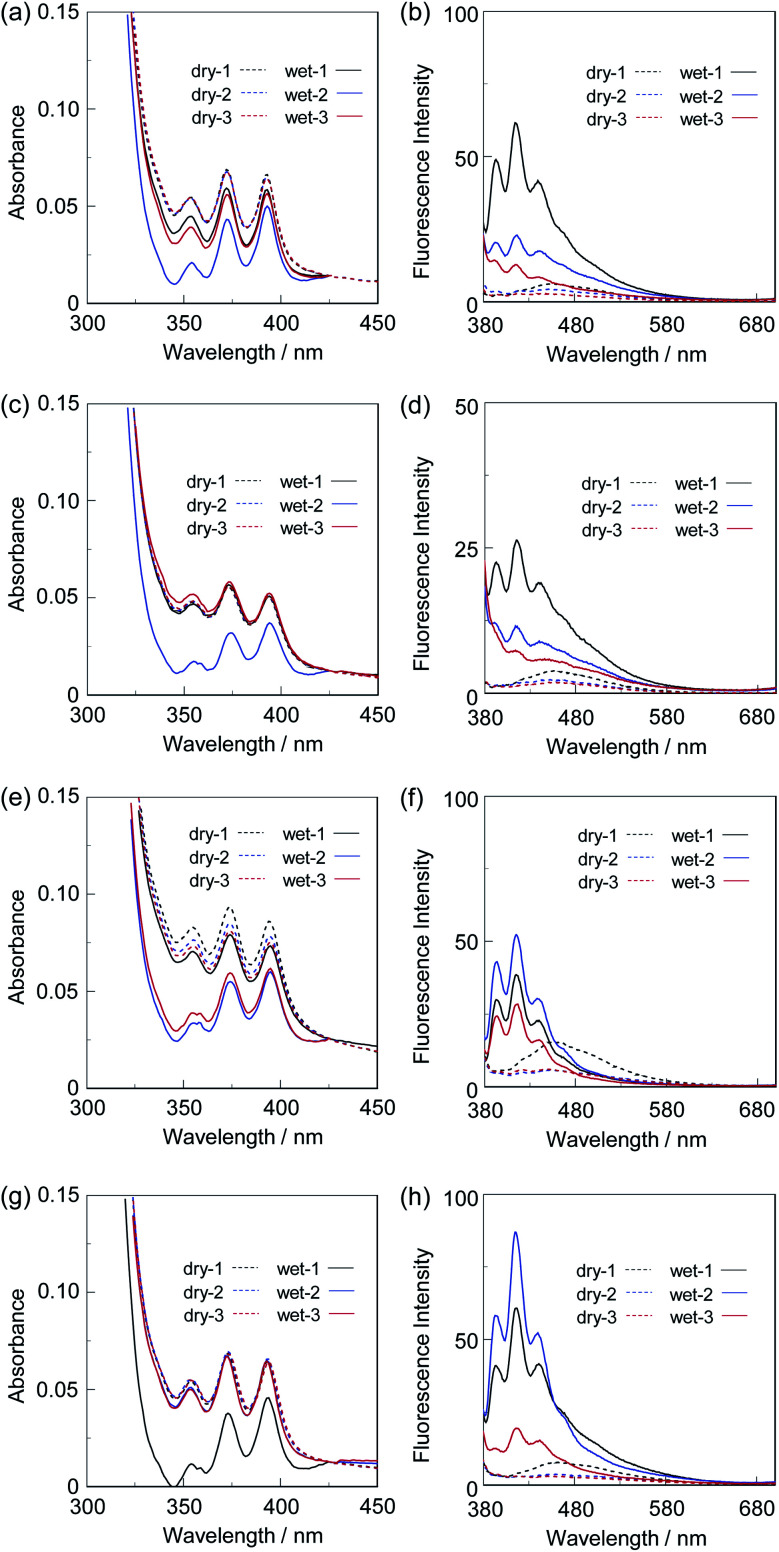
(a) Photoabsorption and (b) fluorescence spectra (*λ*^ex^ = 366 nm) of spin-coated PS film with 50 wt% OF-2 before (in dry process) and after (in wet process) exposure to moisture. (c) Photoabsorption and (d) fluorescence spectra (*λ*^ex^ = 366 nm) of spin-coated PVP film with 50 wt% OF-2 before (in dry process) and after (in wet process) exposure to moisture. (e) Photoabsorption and (f) fluorescence spectra (*λ*^ex^ = 366 nm) of spin-coated PVA film with 50 wt% OF-2 before (in dry process) and after (in wet process) exposure to moisture. (g) Photoabsorption and (h) fluorescence spectra (*λ*^ex^ = 366 nm) of spin-coated PEG film with 50 wt% OF-2 before (in dry process) and after (in wet process) exposure to moisture. For photoabsorption spectra, baseline-correction has been made to be the same absorbance at 425 nm for all the spectra.

**Fig. 5:**
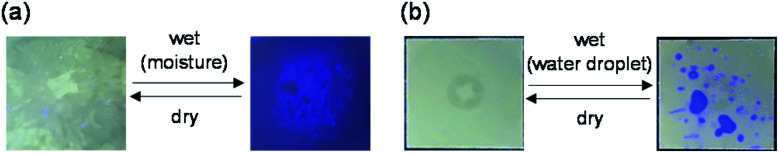
Photographs (under 254 nm irradiation) of 50 wt% OF-2-doped PS film before and after (a) exposure to moisture and (b) water droplet.

**Fig. 6:**
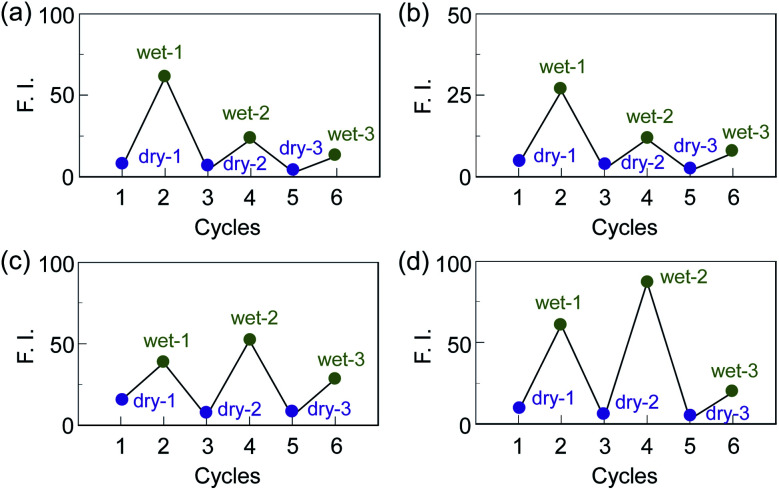
Reversible switching of fluorescence intensity at around 470 nm in dry process and at 415 nm in wet process of 50 wt% OF-2-doped (a) PS, (b) PVP, (c) PVA, and (d) PEG films.

Next, we prepared spin-coated PS films doped with various concentrations (20, 50, and 80 wt%) of OF-2 and investigated the optical sensing properties of the OF-2-doped PS films for water. As with the case of the spin-coated OF-2 film, the as-prepared OF-2-doped PS films (in dry process) show a vibronically-structured photoabsorption band in the range of 300 nm to 400 nm and a feeble and broad fluorescence band in the range of 400 nm to 600 nm attributable to the excimer emission originating from the anthracene skeleton in the PET active state (Fig. 3). With increasing the concentration of OF-2 in the PS films, the absorbance increased, but the intensity of the excimer emission band changed little. When the OF-2-doped PS films were exposed to moisture (in wet process), the photoabsorption spectral shape did not undergo appreciable changes, whereas the fluorescence spectra underwent a change in the spectral shape to the vibronically-structured monomer emission (*λ*^fl^_max_ = 415 nm) arising from the PET inactive state, as with the case of the spin-coated OF-2 film. In addition, for the OF-2-doped PS films after exposure to moisture, the intensity of the monomer emission band increased with the increase in the concentration of OF-2 in the PS films, that is, the fluorescence intensity showed a good linearity as a function of the concentration of OF-2 in the PS films (Fig. 3b inset). Furthermore, we prepared various types of polymer films (PS, PVP, PVA, and PEG) which are doped with OF-2 at 50 wt%, and the photoabsorption and fluorescence spectra of the OF-2-doped polymer films before and after exposure to moisture were repeatedly measured several times (Fig. 4). For all the four OF-2-doped polymer films during the repeated cycles, the photoabsorption spectral shape did not undergo appreciable changes, although a slight change in absorbance was observed due to the disturbance of baseline in the photoabsorption spectra. The corresponding fluorescence spectra show a change in spectral shape from the feeble and broad excimer emission observed in the range of 400 nm to 600 nm to the vibronically-structured monomer emission (*λ*^fl^_max_ = 415 nm) before and after exposure to moisture. In fact, one can see that an as-prepared OF-2-doped PS film initially exhibits the green excimer emission in the PET active state, but the blue monomer emission in the PET inactive state upon exposure to moisture or by water droplet (Fig. 5a and b, see Movie S1 for water droplet, ESI†). The dry–wet cycles of the OF-2-doped polymer films show that a reversible switching in fluorescent intensity between the excimer and monomer emissions was still observed in the third dry–wet process (Fig. 6). Thus, this result demonstrates that the reversibility of the fluorescence intensity of the OF-2-doped polymer films between the excimer and monomer emissions is superior to that of the spin-coated OF-2 film (Fig. 2b inset). On the other hand, a comparison of the reversibility of the fluorescence intensity between the four types of polymer films showed that the hydrophobic PS and PVP films exhibit a good reversibility for moisture from the second dry–wet process, and the hydrophilic PVA and PEG films produce a large change in the intensity between the excimer and monomer emissions during the dry–wet process. Consequently, it was found that PET-type fluorescent sensor-doped polymer films exhibit a reversible switching between the excimer and monomer emissions during the dry–wet process, that is, enable the visualization and detection of moisture and water droplet.

## Conclusions

We have developed polymer films doped with a PET-type fluorescent sensor for water, and demonstrated that the sensor-doped polymer films exhibit a reversible switching in fluorescent color between the green excimer emission in the PET active state under a drying process and the blue monomer emission in the PET inactive state upon exposure to moisture or by water droplet. Thus, we believe that polymer films doped with PET-type fluorescent sensors for water based on a fluorescence enhancement (turn-on) system are one of the most promising and convenient functional materials for visualizing the virus-containing droplet on the surfaces of face shields and partitions.

## Experimental

### General

Photoabsorption spectra of solutions and films were observed with a SHIMADZU UV-3150 spectrophotometer. Fluorescence spectra of solutions, films, and solids were measured with a HITACHI F-4500 spectrofluorometer.

### Preparation of OF-2-doped polymer films

Polystyrene (PS) (2–8 mg) was dissolved in a THF solution (1 mL) of OF-2 (2–8 mg) to form a 20 wt%, 50 wt%, or 80 wt% stock solution. Poly(4-vinylphenol) (PVP) or polyethylene glycol (PEG) (5 mg) was dissolved in a THF solution (1 mL) of OF-2 (5 mg) to form a 50 wt% stock solution. On the other hand, a THF solution (0.5 mL) of OF-2 (5 mg) was added to a polyvinyl alcohol (PVA) (5 mg) aqueous solution (0.5 mL) around 50 °C to form a 50 wt% stock solution. To prepare a polymer film, 300 μL of an OF-2-polymer solution was directly spin-coated (3000 rpm for 30 s) on a glass substrate (MIKASA MS-A-100 Opticoat Spincoater). The spin-coated films were dried in air. The resulting OF-2-doped polymer films were exposed to moisture for 60 s using a humidifier.

## Conflicts of interest

There are no conflicts to declare.

## Supplementary Material

RA-011-D1RA02673A-s001

RA-011-D1RA02673A-s002
