# Supplementary material for: Polymer films doped with fluorescent sensor for moisture and water droplet based on photo-induced electron transfer
Source: RSC Adv. 2021 May 10;11(28):17046–50. doi: 10.1039/d1ra02673a (PMC9031300; doi:10.1039/d1ra02673a)
Supplement: RA-011-D1RA02673A-s002 [file RA-011-D1RA02673A-s002.pdf]

Electronic Supplementary Material (ESI) for RSC Advances.
This journal is © The Royal Society of Chemistry 2021

Supplementary Information

Polymer films doped with fluorescent sensor for moisture and water droplet based on photo-induced electron transfer

Takuma Fumoto, Saori Miho, Yuta Mise, Keiichi Imato and Yousuke Ooyama*

Department of Applied Chemistry, Graduate School of Engineering, Hiroshima University, Higashi-Hiroshima, 739-8527, Japan.

Fax: +81 824 24 5494; Tel: +81 824 24 7689; E-mail:yooyama@hiroshima-u.ac.jp

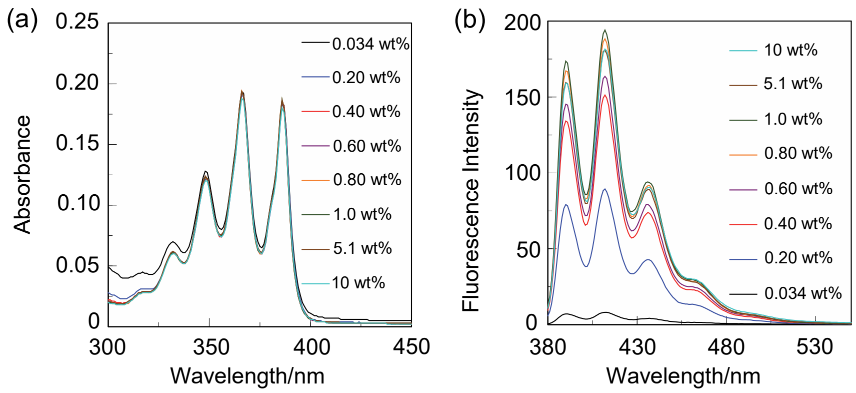

Fig. S1 (a) Absorption and (b) fluorescence spectra (λ^{ex} = 366 nm) of **OF-2** (2.0×10^{-5} M) in acetonitrile containing water (0.034–10 wt%).
